# Genomic Profiling Reveals the Molecular Landscape of Gastrointestinal Tract Cancers in Chinese Patients

**DOI:** 10.3389/fgene.2021.608742

**Published:** 2021-09-14

**Authors:** Chunrong Zhu, Liangjun Zhu, Yanhong Gu, Ping Liu, Xiaoling Tong, Guozhong Wu, Wenyu Zhu, Wenxiang Shen, Hua Bao, Xiangyuan Ma, Ruoying Yu, Xue Wu, Dongqin Zhu, Yongqian Shu, Jifeng Feng

**Affiliations:** ^1^The First Affiliated Hospital of Soochow University, Suzhou, China; ^2^Jiangsu Cancer Hospital, Jiangsu Institute of Cancer Research, Nanjing, China; ^3^Jiangsu Province Hospital, The First Affiliated Hospital of Nanjing Medical University, Nanjing, China; ^4^Nanjing Geneseeq Technology Inc., Nanjing, China; ^5^Wuxi Mingci Hospital, Wuxi, China; ^6^Changzhou No. 2 People’s Hospital Affiliated to Nanjing Medical University, Changzhou, China; ^7^KunShan No.1 People’s Hospital, Kunshan, China; ^8^Jiangsu Provincial Cancer Hospital Affiliated to Nanjing Medical University, Nanjing, China

**Keywords:** gastrointestinal cancers, colorectal cancer, gastric cancer, tumor mutation load, microsatellite instability, chromosomal instability

## Abstract

Gastrointestinal tract cancers have high incidence and mortality in China, but their molecular characteristics have not been fully investigated. We sequenced 432 tumor samples from the colorectum, stomach, pancreas, gallbladder, and biliary tract to investigate cancer-related mutations and detail the landscape of microsatellite instability (MSI), tumor mutation burden (TMB), and chromosomal instability (CIN). We observed the highest TMB in colorectal and gastric cancers and the lowest TMB in gastrointestinal stromal tumors (GISTs). Twenty-four hyper-mutated tumors were identified only in colorectal and gastric cancers, with a significant enrichment of mutations in the polymerase genes (*POLE*, *POLD1*, and *POLH*) and mismatch repair (MMR) genes. Additionally, CIN preferentially occurred in colorectal and gastric cancers, while pancreatic, gallbladder, and biliary duct cancers had a much lower CIN. High CIN was correlated with a higher prevalence of malfunctions in chromosome segregation and cell cycle genes, including the copy number loss of *WRN*, *NAT1*, *NF2*, and *BUB1B*, and the copy number gain of *MYC*, *ERBB2*, *EGFR*, and *CDK6*. In addition, *TP53* mutations were more abundant in high-CIN tumors, while *PIK3CA* mutations were more frequent in low-CIN tumors. In colorectal and gastric cancers, tumors with MSI demonstrated much fewer copy number changes than microsatellite stable (MSS) tumors. In colorectal and gastric cancers, the molecular characteristics of tumors revealed the mutational diversity between the different anatomical origins of tumors. This study provides novel insights into the molecular landscape of Chinese gastrointestinal cancers and the genetic differences between tumor locations, which could be useful for future clinical patient stratification and targeted interventions.

## Introduction

Gastrointestinal (GI) tract cancer refers to a group of cancers affecting the GI and accessory digestive organs, such as the pancreas, liver, gallbladder, and biliary ducts. GI cancers account for almost 30% of all cancer incidences, and 38% of global cancer-related mortality (Bray et al., 2018). GI cancers are difficult to diagnose at early stages due to the lack of symptoms, resulting in limited treatment options for such patients. In the past few years, extensive efforts have been made toward the molecular characterization of GI cancers for the development of novel diagnostic and treatment strategies (Cancer Genome Atlas Network, 2012; Cristescu et al., 2015; Guinney et al., 2015; Liu et al., 2018).

Driver mutations in *TP53*, *APC*, *KRAS*, *BRAF*, and *PIK3CA* are recurrent in GI cancers, but the mutation frequencies vary between different tumor types (Cancer Genome Atlas Network, 2012; Cancer Genome Atlas Research Network, 2014, 2017; Wardell et al., 2018). Genome-level evaluations revealed distinct genomic statuses in GI cancers, including in terms of genomic stability (GS), chromosomal instability (CIN), and microsatellite instability (MSI) that potentially facilitate clinical treatment options (Cancer Genome Atlas Research Network, 2014; Liu et al., 2018). CIN is defined as whole-chromosome mis-segregation that results in the loss or gain of large chromosomal fragments, and is positively correlated to tumor metastasis, poor prognosis, and treatment resistance (Lee et al., 2011; Pikor et al., 2013; Bakhoum and Cantley, 2018). Conversely, MSI, which is characterized as high numbers of mutations in microsatellite repeats, is associated with increased intra-tumor immune infiltration and better prognoses (Pages et al., 2005, 2008). Thus, newly identified tumor biomarkers are significantly changing the tumor staging systems and treatment landscapes for GI cancers.

In China, stomach and colorectal cancers are the leading causes of death, immediately after lung and liver cancers (Chen et al., 2016). However, pancreatic, gallbladder, and biliary duct cancers are relatively rare, but their prognoses are much worse, and the availability of non-surgical treatments is limited. Compared to Western countries, the high smoking rate, the prevalence of *Helicobacter pylori* infections, heavy alcohol consumption, and poor nutrition are the factors contributing to the high incidences of digestive system cancers in China (Gu et al., 2018). Here, we report the molecular profiles of 423 GI tumors using targeted gene sequencing, including those of gastric cancer (GAST), colorectal cancer (CORE), pancreatic cancer (PAAD), gallbladder and biliary tract cancer (GABI), and gastrointestinal stromal tumors (GISTs). Recurrent somatic mutations and copy number variations (CNVs) were identified and compared across different tumors or sub-locations of the same tumors. The tumor genomes were also detailed for MSI, tumor mutation burden (TMB), and chromosome instability (CIN) that are closely related to treatment selection and prognostic predictions.

## Materials and Methods

### Patient Recruitment and Tumor Sample Collection

The study cohort was identified from several key cancer hospitals in Jiangsu province, China. All patients were diagnosed with GAST, CORE, PAAD, GABI, or GIST between February 2015 and February 2017, and submitted tumor tissues for clinical tumor genetic testing to assist with clinical decision-making. Clinical information and the genetic testing data of these patients were retrospectively collected from their registration forms. TNM status of each patient was defined at the time of genetic sequencing, rather than at the initial diagnosis. Primary tumor sites were used to divide tumors into different sub-locations for cross-comparisons. For colorectal cancer, right-sided tumors included tumors in the proximal two-thirds of the transverse colon, ascending colon, and cecum, while left-sided tumors included tumors in the distal one-third of the transverse colon, descending colon, and rectum.

For each patient, formalin-fixed paraffin-embedded (FFPE) and matched whole blood samples were submitted for targeted next-generation sequencing using a customized panel of 416 cancer-related genes, as described in previous reports (Yang et al., 2018). The study methodologies conformed to the standards set by the Declaration of Helsinki and were approved by the Ethics Committee of Jiangsu Province Cancer Hospital. All patients provided informed written consent. All samples were tested in a certified genomic testing facility (Nanjing Geneseeq Technology Inc., Nanjing, China).

### DNA Library Preparation and Next-Generation Sequencing

DNA extraction was performed using the same protocols as in our previous publications (Shu et al., 2017; Yang et al., 2018). In brief, FFPE DNA was purified using the QIAamp DNA FFPE Tissue Kit (Qiagen, Hilden, Germany) and genomic DNA from white blood cells was extracted using the DNeasy Blood & Tissue Kit (Qiagen), following the manufacturer’s protocols. DNA samples were quantified using the dsDNA HS Assay Kit on a Qubit 3.0 Fluorometer (Life Technologies, Carlsbad, CA, United States).

The KAPA Hyper Prep Kit was used to prepare sequencing libraries (KAPA Biosystems, Wilmington, MA, United States), as described previously (Yang et al., 2018). Libraries were then PCR amplified and purified before target enrichment. As described previously (Yang et al., 2018), during enrichment, indexed DNA libraries were pooled to up to 2 μg of total input and then subjected to hybridization capture of the targeted gene regions using custom DNA probes (Integrated DNA technologies, San Jose, CA, United States). After target enrichment, libraries were sequenced on the HiSeq4000 platform (Illumina, San Diego, CA, United States) with 2 × 150 bp pair-end reads.

### Data Processing

Sequenced reads were analyzed by Trimmomatic (Bolger et al., 2014) to remove low-quality (quality < 15) or N bases and mapped to the human reference genome Human Genome version 19 (hg19) using the Burrows-Wheeler Aligner (BWA) (Li and Durbin, 2009). Picard was used to remove PCR duplicates, and the cutoff for qualified sequences of the tumor tissues was a mean coverage depth of >100 × after removing PCR duplicates. The Genome Analysis Toolkit (GATK) was used to perform local realignments around insertions/deletions (indels), base quality reassurance, and the discovery of germline variations (DePristo et al., 2011). Somatic single nucleotide variants (SNVs) and small indels were called by VarScan2 (Koboldt et al., 2012) and HaplotypeCaller/UnifiedGenotyper in GATK. Somatic variant calls with at least a 1.0% mutant allele frequency (MAF) and with at least three supporting reads in both directions were retained. Common variants were removed using dbSNP and the 1000 Genome project. Annotation was performed using ANNOVAR (Wang et al., 2010). Gene fusions were identified by FACTERA (Newman et al., 2014) and manually inspected using the Integrative Genomics Viewer (IGV). To identify somatic CNVs, CNVkit was used to analyze segmentations (Amarasinghe et al., 2013) and the results were fed into the GISTIC algorithm to identify recurrent focal and arm-level CNVs with a cutoff *q*-value of 0.25 (Mermel et al., 2011). TMB was determined based on the number of somatic base substitutions and indels in the targeted regions of the gene panel covering 1.2 Mbp of coding genome. In agreement with previous publications (Goodman et al., 2017), hyper-mutated tumors were defined as tumors with a TMB of >20 mutations/Mb (Supplementary Figure 2). Actionable mutations were defined by the Database of Evidence for Precision Oncology (DEPO) (Sun et al., 2018), including missense, in-frame and frameshift indels, splice site variations, and stop-gain mutations.

The MSI of each sample was determined by evaluating 52 embedded mononucleotide repeats with a minimum of 15-bp repeats that were included in the sequencing panel. The baseline length distribution of each repeat was determined from a pool of microsatellite-stable samples. A sample was identified as MSI if more than 45% of the qualified sites displayed instability.

The data from genome segments inferred by CNVkit were used to analyze the CIN score, which was defined as the proportion of the genome with aberrant segmented copy numbers. DNA segments with a log2 ratio below −0.2 or above 0.2 were considered as exhibiting copy number variance (CNV). The proportion of such segments in all of the covered regions of the genome was calculated as the CIN score. High CIN samples were defined as the upper 25% in all tumors, while low CIN samples were defined as the lower 25% in all tumors.

## Results

### Clinical Characteristics of the Enrolled Patients and Samples Submitted for Sequencing

All 432 patients had at least one qualified tumor sample submitted for targeted NGS, and 18 individuals were excluded from the final analysis due to the lack of mutations in any of their tumor samples (Supplementary Figure 1). Finally, 414 patients, including CORE (*n* = 207), GAST (*n* = 144), PAAD (*n* = 27), GABI (*n* = 14), and GIST (*n* = 22) had adequate tumor tissue samples sequenced and were analyzed for somatic missense mutations, small indels, CNVs, and chromosomal rearrangements. The clinical characteristics of these patients are summarized in Supplementary Tables 1–5.

### Different Cancers Exhibited Distinct Prevalence of Somatic Gene Alterations

**FIGURE 1:**
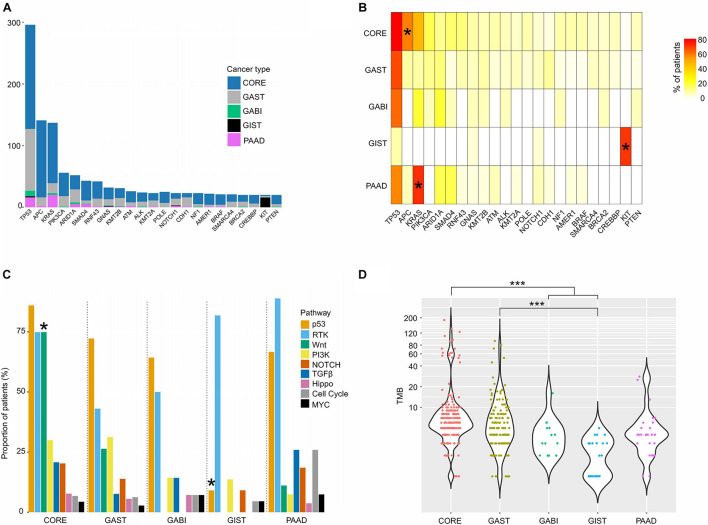
Somatic mutations of different GI cancers. **(A)** Bar graph showing the patient composition of commonly mutated genes. **(B)** The mutation frequency of the top mutated genes in each tumor. **(C)** The proportion of patients with mutations in each pathway. **(D)** TMB distribution of each cancer. For **(B,C)**, pairwise comparisons were conducted between every two groups using the Fisher’s exact test. FDR was used for *p*-value corrections. For **(D)**, one-way ANOVA on ranks test was used to compare all groups, and the Dunn’s test was used for *post hoc* analyses. *p < 0.05; ***p < 0.001.

An overview of the top somatic mutations (mutation frequency >5%) in all tumors revealed highly recurrent mutations in *TP53* (*n* = 296, 71% of all patients), *APC* (*n* = 141, 34%), and *KRAS* (*n* = 137, 33%), and the mutation frequencies vary greatly between different cancers (Figure 1A). For example, *TP53* was commonly altered in CORE (82%), GAST (70%), GABI (64%), and PAAD (59%), but rarely in GIST (9%, Supplementary Figure 3). *APC* was mutated significantly more frequently in CORE (60%), while *KRAS* was specifically enriched in PAAD (74%, Supplementary Figure 3A) and CORE (50%, Figure 1B). Moreover, 73% of GIST had alterations in *KIT*, the majority of which were indels in exon 11 (Supplementary Figure 3B). This finding was consistent with previous reports (Debiec-Rychter et al., 2006; Wozniak et al., 2012).

Key cancer-associated genes covered by the sequencing panel were classified into nine canonical signaling pathways responsible for cellular proliferation, as previously described (Sanchez-Vega et al., 2018; Supplementary Table 6). We computed the fraction of samples with at least one gene altered in each pathway, and found that p53, RTK-RAS, and Wnt were the most frequently mutated pathways, while MYC, cell cycle, and Hippo pathways had the lowest mutation ratio in nearly all cancers (Figure 1C). The Wnt pathway was mutated more frequently in CORE (75%) than in other cancers (*p* < 0.05), and the RTK pathway was altered extensively in both GIST (82%) and PAAD (89%). For the Wnt pathway, apart from *APC* (60% of CORE), other genes, including *RNF43* (14%), *AMER1* (9%), *AXIN2* (6%), *CTNNB1* (5%), *CHD4* (4%), and *LZTR1* (4%), were also mutated in CORE with different frequencies, which raises therapeutic opportunities by inhibiting the Wnt pathway. GIST had the highest mutation frequency of the RTK pathway, and the lowest mutation frequency of the p53 pathway (9%, *p* < 0.05) among all cancer types (Figure 1C). In the RTK pathway, *KIT* was the most frequently altered gene in GIST (73%), while *KRAS* alterations were predominantly found in CORE (47%), GAST (11%), GABI (21%), and PAAD (74%).

When stratifying all patients based on the existence of actionable mutations, 47% of all cases harbored at least one actionable mutation (Supplementary Table 7). The highest frequencies were found in PAAD (77%) and GIST (77%) due to the extensive mutations in *KRAS* and *KIT* (Supplementary Figure 4A). A total of 56% of CORE cases and 24% of GAST cases were defined as actionable, with hotspot mutations at *KRAS* p.G12/G13/Q61 and *PIK3CA* p.E542/E545/H1047R being the most dominant (Supplementary Figure 4B). In addition, *BRAF* p.V600E, p.G466V, and p.L597R, as well as *BRCA2* mutations, were also considered actionable based on the observations from other tumor types (Gautschi et al., 2015; Robert et al., 2015; Pujade-Lauraine et al., 2017).

### Tumor TMB and CIN Indicate New Treatment Strategies

**FIGURE 2:**
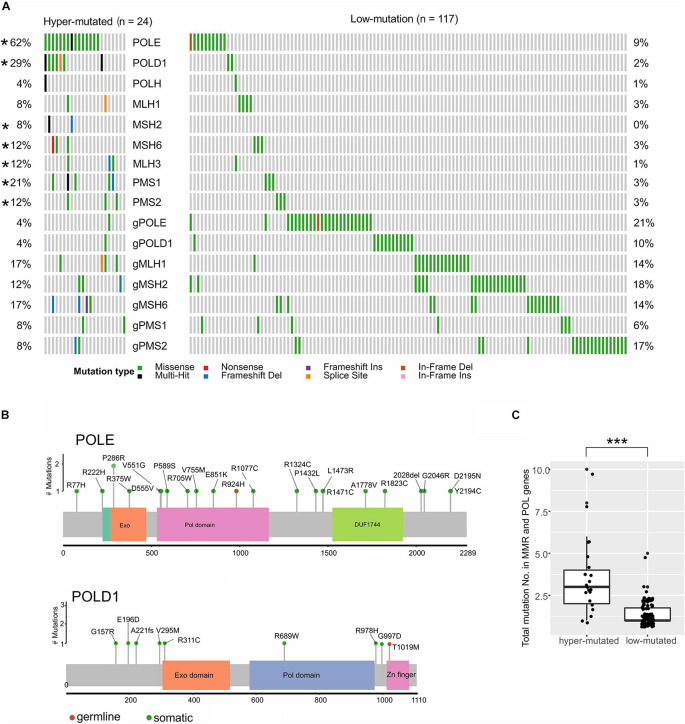
MMR and polymerase genes showed different mutation frequencies in low-mutated and hyper-mutated tumors. **(A)** Somatic and germline mutations in MMR and polymerase genes. “g” represents germline mutations; *p < 0.05. **(B)** The lollipop plot shows the scattered amino acid changes in POLE and POLD1. **(C)** The mutation number of MMR and polymerase genes in each patient; ***p < 0.001.

The TMB of all patients ranged between 1 and 185 (median: 5), with the highest median in CORE (median: 6) and the lowest median in GIST (median: 2.5, Figure 1D). However, both CORE (9%) and GAST (3%) had a small high-TMB population, named hyper-mutated tumors in this study (*n* = 26, Figure 1D and Supplementary Figure 2). Of these 24 hyper-mutated tumors, 23 (92%) had at least one somatic or germline mutation in the MMR genes, including *MLH1*, *MSH2*, *MSH6*, *PMS1*, and *PMS2*, or the polymerase (POL) genes, including *POLE*, *POLD1*, and *POLH.* In low-mutation tumors, only 30% of tumors (117 out of 388 tumors) had mutations in the MMR and POL genes (Figure 2A). Somatic mutations in *MSH2*, *MSH6, MLH3, PMS1*, *PMS2*, *POLE*, and *POLD1* were significantly higher in hyper-mutated tumors than low-mutation tumors (FDR < 0.01). *POLE* and *POLD1* mutations (including somatic and germline) in hyper-mutated tumors were dispersed across all domains of the two POLs (Figure 2B), with only *POLE* p.P286R and *POLD1* p.R689W being reported to have functional disruption (Ahn et al., 2016; Mertz et al., 2017). However, we observed that a *POLE* p.A1778V mutation in patient #GA_59, who developed gastric cancer at age 76 with a TMB of 45 (71% were missense mutations), was the only mutation in the polymerases and MMR genes in this patient, suggesting that this mutation might impair POLE function. We also identified a novel mutation in *POLD1* from three hyper-mutated patients (#CO_129, CO_26, and CO_273), a somatic splicing variant c.2954-1delG that disrupts the zinc finger domain of POLD1 and can be potentially harmful. Notably, in low-mutation tumors, somatic and germline mutations in the MMR and POL genes were almost exclusive from each other, while in the hyper-mutated group, the total number of mutations in the MMR and POL genes were significantly higher (*p* < 0.01, Figure 2C).

**FIGURE 3:**
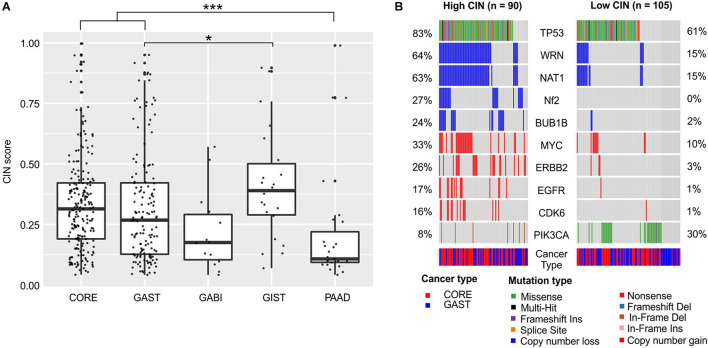
Chromosome instability in each cancer and its association with gene alterations. **(A)** The distribution of CIN scores in each cancer. **(B)** Gene mutations and copy number changes that were significantly different between the high-CIN and low-CIN groups. For **(A)**, the one-way ANOVA on ranks test was used to compare all groups, and the Dunn’s test was used for *post hoc* analyses. *p < 0.05; ***p < 0.001. For **(B)**, pairwise comparisons were conducted between every two groups using the Fisher’s exact test. FDR was used for *p*-value correction.

Another genome marker that we inspected was CIN. CIN is a critical hallmark of cancer and is closely related to tumor metastasis, treatment resistance, and poor prognosis (Pikor et al., 2013; Bakhoum and Cantley, 2018). The CIN score was used to measure the extent of copy number changes in large segments in an individual tumor. The CIN score ranged widely in each cancer (Figure 3A). The median CIN was relatively higher in GIST (0.40), CORE (0.31), and GAST (0.27) tumors, and lower in GABI (0.18) and PAAD (0.11) tumors (Figure 3A). The mechanisms causing CIN have not been fully elucidated. It was suggested that chromosome segregation genes and cell cycle genes were widely related to CIN (Maleki and Rocken, 2017).

We compared the mutation frequencies between high-CIN and low-CIN tumors to identify the associated gene alterations (Figure 3B). *TP53* was significantly enriched in high-CIN tumors (FDR < 0.01), which was consistent with previous reports of mitotic stress caused by *TP53* malfunctions (Malumbres, 2011). In addition, we also observed broad copy number loss of *WRN*, *NAT1*, *NF2*, and *BUB1B*, as well as copy number gain of *MYC*, *ERBB2*, *EGFR*, and *CDK6* in high-CIN tumors (FDR < 0.01, Figure 3B). The copy number loss of *WRN* and *NAT1* were almost concurrent, possibly because of their adjacent genomic locations. *PIK3CA* is the only signature that was significantly enriched in low-CIN tumors (FDR < 0.1, Figure 3B).

### Colorectal and Gastric Cancers Showed Location-Specific Gene Alterations

**FIGURE 4:**
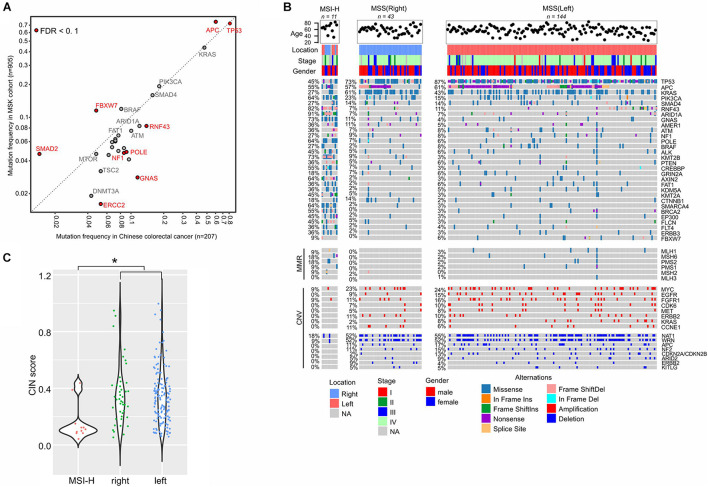
Molecular characterization of colorectal cancer. **(A)** Mutation frequency comparison between our cohort and the MSKCC colorectal cancer cohort. **(B)** Gene alterations in the sub-groups of colorectal cancers. **(C)** CIN status in different sub-groups. Dunn’s multiple comparison test is used to compare CIN values between every two groups and * means *p* < 0.05.

In order to investigate the interethnic differences, we compared the prevalence of somatic mutations between our colorectal cancer cohort (*n* = 207) and the Memorial Sloan Kettering Cancer Center (MSKCC) metastatic colorectal cohort (*n* = 985) (Yaeger et al., 2018). The two groups had comparable clinical features with respect to patients’ ages, gender, and the primary tumor locations. However, our cohort had more stage IV disease patients, at 80.3% vs. 61.7% in MSKCC cohort (Supplementary Table 1). *TP53* was the most frequently mutated gene in both cohorts, but in our cohort, the mutation ratio was significantly higher (81% vs. 73%, FDR = 0.02), which is consistent with its presence in more advanced diseases (Yaeger et al., 2018). Conversely, *FBXW7*, whose mutations were suggested to be enriched in early-stage tumors (Yaeger et al., 2018), was mutated less in our cohort (Figure 4A). We also found that *APC* alterations were less frequent in our cohort (60% vs. MSKCC 75%, FDR = 0.0007), while another Wnt pathway driver, *RNF43*, was more frequently mutated (14% vs. MSKCC 8%, FDR = 0.07). Other genes that were increasingly mutated in our cohort were *GNAS* (11%), *POLE* (9%), *NF1* (9%), and *ERCC2* (5%), while *SMAD2* (1%) was mutated significantly less frequently (FDR < 0.1).

We classified our cohort into highly microsatellite-instable (MSI-H, *n* = 11) and microsatellite-stable (MSS) tumors given their MSI status identified by the embedded microsatellite sequences in the targeted sequencing panel (Figure 4B). The MSS group was further divided into right-sided (*n* = 48) and left-sided (*n* = 149) tumors for comparison. The incidence of the left-sided MSS tumors (72%, *n* = 149) was much higher than the right-sided MSS tumors (22%, *n* = 48), while 10 cases were without tumor location information. As expected, DNA mismatch repair (MMR) genes were more frequently mutated in MSI-H tumors, compared to MSS tumors. In the MSI-H group, we observed an enrichment of somatic mutations in a number of genes, and the top affected genes were *ARID1A* (mutation frequency: 91%), *RNF43* (82%), *GNAS* (73%), *KMT2B* (73%), *PIK3CA* (64%), *POLE* (64%), *AXIN2* (64%), and *SMARCA4* (64%), because of the existence of short tandem repeats in gene sequences that can be easily affected by MMR gene defects. However, CNVs were scarcely observed in MSI-H tumors (Figure 4B).

In MSS tumors, gene alterations were imbalanced between the right side and left side. The left-sided tumors were characterized by higher levels of *TP53* mutations (87%), while the right-sided tumors exhibited higher levels of *KRAS* (43%) and *CTNNB1* (2%) mutations (FDR < 0.1). Meanwhile, copy number loss of *CDKN2A/CDKN2B* was significantly more prevalent in right-sided tumors (Figure 4B, 13% vs. 2% in left-sided tumors, FDR < 0.1). The degree of CIN in MSI-H tumors (median of CIN score: 0.11) was significantly lower than in MSS tumors (median 0.32, *p* = 0.0036), and the scores were similar between the right-sided and left-sided tumors (Figure 4C).

**FIGURE 5:**
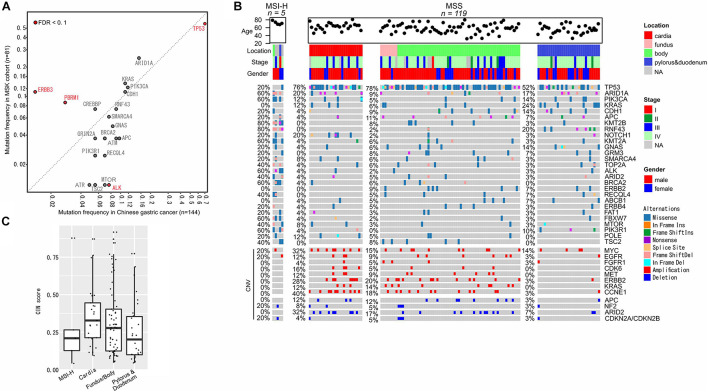
Molecular characterization of gastric cancer. **(A)** Mutation frequency comparisons between our cohort and the MSKCC gastric cancer cohort. **(B)** Gene alterations in the sub-groups of colorectal cancers. **(C)** CIN status in different sub-groups.

The prevalence of gene alterations in gastric cancer was also compared to the MSKCC cohort (*n* = 81), and revealed a higher mutation frequency in *TP53* and lower mutation frequency in *PBRM1* and *ERBB3* (Figure 5A). Similar to colorectal cancer, gastric cancer was first classified into MSI-H (*n* = 5) and MSS (*n* = 119) cases, and then MSS cases were further grouped based on the locations of primary tumors, including cardia (*n* = 25), fundus and body (*n* = 65), and pylorus and duodenum (*n* = 29). As expected, MSI-H cases showed markedly increased frequencies of somatic mutations and decreased CNVs compared to MSS cases (Figure 5B). *RNF43* and *KRAS* mutations were primarily observed in pylorus duodenum regions, while *CCNE1* amplifications were prevalent in the upper portion of the stomach (cardia, fundus, and body). *KRAS* and *ERBB2* mutations were not present in any of the MSI-H patients (Figure 5B). Consistent with a previous report (Cancer Genome Atlas Research Network, 2014), the cardia section of the stomach has relatively high CIN scores and the median is gradually reduced from the upper stomach to the bottom stomach (Figure 5C).

## Discussion

Herein, we performed a comprehensive genetic analysis of different GI cancers. This is the first large-scale study of GI cancers in Chinese patients that implemented a uniform genetic testing and data analysis pipeline. The results highlighted the similarities and differences in the genetic landscapes of GI cancers and also informed on the status of several biomarkers for cancer treatment, including MSI status, TMB, and CIN between different cancer types. The high mutation frequency of *TP53*, *APC*, and *KRAS* in CORE and that of *KRAS* in PAAD were reported in multiple other studies (Vogelstein et al., 1988; Kinzler and Vogelstein, 1996; Bardeesy and DePinho, 2002). However, the frequency of *TP53* alterations was higher in our CORE cohort than in other reported populations (Olivier et al., 2002; Abubaker et al., 2008). Such a finding might be due to the presence of more advanced disease stages at the time of diagnosis in this study. Mutations in the *KIT* gene were recognized as a relatively early event in GIST tumorigenesis, while *TP53* mutations were related to the malignant transformation of GIST (Ryu et al., 2004).

Although we lacked pathological stage information of the GIST patients in our cohort, the high *KIT* mutation frequency and low *TP53* mutation frequency that we observed suggested that GIST patients were at an early disease stage. This assumption was also supported by the fact that we did not observe high frequencies of *RB1* mutations in the GIST population, which was an event that might be restricted to malignant GISTs (Merten et al., 2016).

Significantly aberrant Wnt signaling was observed in CORE compared to other cancer types, and this pathway has been closely linked to carcinogenesis (Mirabelli et al., 2019). Many inhibitors targeting the Wnt signaling pathway are being examined in different clinical trials, including porcupine (PORCN) inhibitors, WNT ligand antagonists, and FZD antagonists/monoclonal antibodies (Jung and Park, 2020). In Chinese CORE and GAAD patients, both *APC* and *RNF43* were predominantly mutated in the Wnt pathway. A significant subset of patients had nonsense or frameshift alterations in *RNF43*, particularly high frequencies in the MSI-H group. Such mutations were also mutually exclusive with *APC* alterations. As a tumor suppressor, *RNF43* has shown its capacity to negatively regulate Wnt signaling (Koo et al., 2012; Loregger et al., 2015). Recent studies found that depletion of RNF43 enhanced tumor growth in GI cancers and conferred resistance to DNA-damage-inducing chemotherapies and γ-radiation in gastric cancer cells (Neumeyer et al., 2019, 2020). Additionally, preclinical cancer models have shown the responsiveness of *RNF43* mutations to Wnt inhibitors, several of which are in clinical trials (Janku et al., 2015, 2020; Yu et al., 2020). Therefore, screening for *RNF43* mutational status could direct therapy selections for GI cancer treatments.

Among all cancer types, CORE and GAST demonstrated significantly higher TMB than others, while GIST demonstrated relatively higher CIN scores. In both CORE and GAST, a small group of patients were characterized by MSI-H, and their CIN levels were correspondingly lower than those of the MSS groups, thus suggesting that tumors obtain a survival advantage through either high mutational loads or high levels of somatic copy number alterations (SCNA). Both TMB and MSI are emerging biomarkers for immune checkpoint inhibitors and CIN has the potential to drive tumor evolution and treatment resistance (Jin et al., 2020). CIN, which was characterized by increasing the mis-segregation of chromosomes, can be induced by defects in the mitotic spindle assembly checkpoints, cell cycle regulation, multipolar spindles, or DNA damage responses (Thompson et al., 2010; Bakhoum et al., 2012). The acquisition of CIN is an essential feature in cancer pathogenesis and is considered as compensation for a lack of driver mutations (Turajlic et al., 2018). CIN is also considered a drug-resistant mechanism during cancer treatment and negatively correlates with the progression-free survival and overall survival of cancer patients (Turajlic et al., 2018; Jin et al., 2020). In our cohort, we observed a high level of *TP53* mutations in high-CIN patients, while *PIK3CA* alterations were significantly enriched in low-CIN patients, with a tendency for mutual exclusivity with *TP53* mutations. These findings are consistent with previous reports that *TP53* inactivation results in CIN tolerance in cells (Thompson and Compton, 2010; Matano et al., 2015).

Although *PIK3CA* acts independently of *TP53* inactivation to support CIN tolerance, it generally precedes the genome doubling event (Carter et al., 2012; Zack et al., 2013; Berenjeno et al., 2017). The GIST population has the highest median level of CIN, despite its low mutation frequency in *TP53* and the cell cycle pathway compared to that observed in other cancer types. A further look at the high-CIN and median-CIN groups of GIST identified a much higher level of *NF2* copy number deletion in the high-CIN group than the low-CIN group. NF2 inactivation has been linked to increased CIN in meningiomas (Goutagny et al., 2010; Dewan et al., 2017), but for the first time, we report that its copy number deletion is potentially associated with high CIN level in GIST. However, this finding must be validated in a much larger cohort of GIST samples.

Although CIN potentially drives tumor evolution and drug resistance *via* the production of oncogenic SCNA, excessive levels of CIN were proven to be detrimental to tumor growth (Roylance et al., 2011; Janssen and Medema, 2013), thus creating the opportunity for developing therapies aimed at increasing the CIN level of tumors. Currently, a few agents targeting Mps1/TTK kinase to induce CIN have been evaluated in phase I clinical trials, including BAY1217389 (NCT02366949), BAY1161909 (NCT02138812), and BOS172722 (NCT03328494; clinicaltrials.gov). However, the success of this strategy relies on patient stratification based on their CIN levels, and the co-existence of gene alterations (e.g., *TP53* or *PIK3CA*) that can reduce the toxicity of elevated CIN.

Malfunctioning DNA repair mechanisms caused by somatic mutations in MMR genes is common in cancer and contributes to MMR deficiency, and high TMB and MSI phenotypes (Bodor et al., 2018). Indeed, the majority of the hyper-mutated tumors in our cohort were observed to have somatic mutations in MMR genes. MSI-H tumors were also found to have a higher ratio of MMR gene mutations compared to MSS tumors. Recent studies have suggested that mutations in DNA polymerase (POL) genes are other factors that are associated with a hyper-mutated tumor phenotype, especially in colon and rectal cancers (Cancer Genome Atlas Network, 2012). Interestingly, in hyper-mutated tumors, we observed concomitant somatic or germline mutations in the MMR and POL genes, with a median of three MMR/POL mutations per tumor, which was significantly higher than that of low-mutation tumors (a median of one MMR/POL mutation per tumor). However, currently only a few non-synonymous mutations in the exonuclease domains (EDM) of *POLE* (residues 268–471) and *POLD1* (residues 304–517) have been considered pathogenic (Briggs and Tomlinson, 2013), while most others are classified as variants of unknown significance. Increasing evidence suggests that patients with *POLE* EDMs are prone to higher TMBs and an upregulation of immune checkpoint genes, which could potentially benefit from immune checkpoint inhibitors (Snyder et al., 2014; Rayner et al., 2016; Mo et al., 2020).

The limitations of this study included the lack of clinical treatment and prognostic information, which is typical in any retrospective study. Therefore we are unable to determine the treatment outcomes that were potentially linked to the genomic findings of different cancer types. However, our analysis of a large cohort of advanced GIs revealed the landscape of genetic alterations, highlighted the genomic differences between tumor locations, such as between right- and left-sided CRC, and identified the unique molecular features in Asian GI cancer patients.

## Data Availability Statement

The original contributions presented in the study are included in the article/Supplementary Material, further inquiries can be directed to the corresponding author/s.

## Ethics Statement

The studies involving human participants were reviewed and approved by the Ethics Committee of Jiangsu Province Cancer Hospital. Written informed consent to participate in this study was provided by the participants or their legal guardian/next of kin.

## Author Contributions

JF, PL, CZ, LZ, and YG: conceptualization and critical thinking. XT, GW, HB, XM, RY, and XW: perform experiments and data analysis. CZ, LZ, YG, GW, WZ, WS, and DZ: clinical sample collection and pathological analyses. CZ, LZ, YG, XT, and RY: manuscript drafting. JF, PL, CZ, LZ, YG, XT, RY, and XW: manuscript review and editing. All authors contributed to the article and approved the submitted version.

## Conflict of Interest

XT, HB, XM, RY, XW, and DZ are shareholders or employees Geneseeq Technology Inc. The remaining authors declare that the research was conducted in the absence of any commercial or financial relationships that could be construed as a potential conflict of interest.

## Publisher’s Note

All claims expressed in this article are solely those of the authors and do not necessarily represent those of their affiliated organizations, or those of the publisher, the editors and the reviewers. Any product that may be evaluated in this article, or claim that may be made by its manufacturer, is not guaranteed or endorsed by the publisher.

## References

[B1] Abubaker J. Bavi P. Al-Harbi S. Ibrahim M. Siraj A. K. Al-Sanea N. (2008). Clinicopathological analysis of colorectal cancers with PIK3CA mutations in Middle Eastern population. *Oncogene* 27 3539–3545. 10.1038/sj.onc.1211013 18193083

[B2] Ahn S. M. Ansari A. A. Kim J. Kim D. Chun S. M. Kim J. (2016). The somatic POLE P286R mutation defines a unique subclass of colorectal cancer featuring hypermutation, representing a potential genomic biomarker for immunotherapy. *Oncotarget* 7 68638–68649. 10.18632/oncotarget.11862 27612425 PMC5356579

[B3] Amarasinghe K. C. Li J. Halgamuge S. K. (2013). CoNVEX: copy number variation estimation in exome sequencing data using HMM. *BMC Bioinformatics* 14:S2. 10.1186/1471-2105-14-S2-S2 23368785 PMC3549847

[B4] Bakhoum S. Compton D. Bakhoum S. Compton D. (2012). Chromosomal instability and cancer: a complex relationship with therapeutic potential. *J. Clin. Investig.* 122 1138–1143. 10.1172/jci59954 22466654 PMC3314464

[B5] Bakhoum S. F. Cantley L. C. (2018). The Multifaceted Role of Chromosomal Instability in Cancer and Its Microenvironment. *Cell* 174 1347–1360. 10.1016/j.cell.2018.08.027 30193109 PMC6136429

[B6] Bardeesy N. DePinho R. A. (2002). Pancreatic cancer biology and genetics. *Nat. Rev. Cancer* 2 897–909. 10.1038/nrc949 12459728

[B7] Berenjeno I. M. Pineiro R. Castillo S. D. Pearce W. Mcgranahan N. Dewhurst S. M. (2017). Oncogenic PIK3CA induces centrosome amplification and tolerance to genome doubling. *Nat. Commun.* 8:1773. 10.1038/s41467-017-02002-4 PMC5701070 29170395

[B8] Bodor J. N. Handorf E. A. Feldman R. Hall M. J. (2018). Pathogenic somatic mutation (SM) of mismatch repair (MMR) genes and associations with microsatellite instability (MSI), tumor mutational burden (TMB) and SM in other DNA repair pathways in 24,223 tumor genomic profiles. *J. Clin. Oncol.* 36 1505–1505. 10.1200/jco.2018.36.15_suppl.150529617189

[B9] Bolger A. M. Lohse M. Usadel B. (2014). Trimmomatic: a flexible trimmer for Illumina sequence data. *Bioinformatics* 30 2114–2120. 10.1093/bioinformatics/btu170 24695404 PMC4103590

[B10] Bray F. Ferlay J. Soerjomataram I. Siegel R. L. Torre L. A. Jemal A. (2018). Global cancer statistics 2018: GLOBOCAN estimates of incidence and mortality worldwide for 36 cancers in 185 countries. *CA Cancer J. Clin.* 68 394–424. 10.3322/caac.21492 30207593

[B11] Briggs S. Tomlinson I. (2013). Germline and somatic polymerase epsilon and delta mutations define a new class of hypermutated colorectal and endometrial cancers. *J. Pathol.* 230 148–153. 10.1002/path.4185 23447401 PMC3709119

[B12] Cancer Genome Atlas Network. (2012). Comprehensive molecular characterization of human colon and rectal cancer. *Nature* 487 330–337. 10.1038/nature11252 22810696 PMC3401966

[B13] Cancer Genome Atlas Research Network. (2014). Comprehensive molecular characterization of gastric adenocarcinoma. *Nature* 513 202–209. 10.1038/nature13480 25079317 PMC4170219

[B14] Cancer Genome Atlas Research Network. (2017). Integrated Genomic Characterization of Pancreatic Ductal Adenocarcinoma. *Cancer Cell* 32 185–203.e13. 28810144 10.1016/j.ccell.2017.07.007 PMC5964983

[B15] Carter S. L. Cibulskis K. Helman E. Mckenna A. Shen H. Zack T. (2012). Absolute quantification of somatic DNA alterations in human cancer. *Nat. Biotechnol.* 30 413–421. 10.1038/nbt.2203 22544022 PMC4383288

[B16] Chen W. Zheng R. Baade P. D. Zhang S. Zeng H. Bray F. (2016). Cancer statistics in China, 2015. *CA Cancer J. Clin.* 66 115–132. 10.3322/caac.21338 26808342

[B17] Cristescu R. Lee J. Nebozhyn M. Kim K. M. Ting J. C. Wong S. S. (2015). Molecular analysis of gastric cancer identifies subtypes associated with distinct clinical outcomes. *Nat. Med.* 21 449–456. 25894828 10.1038/nm.3850

[B18] Debiec-Rychter M. Sciot R. Le Cesne A. Schlemmer M. Hohenberger P. Van Oosterom A. T. (2006). KIT mutations and dose selection for imatinib in patients with advanced gastrointestinal stromal tumours. *Eur. J. Cancer* 42 1093–1103. 10.1016/j.ejca.2006.01.030 16624552

[B19] DePristo M. A. Banks E. Poplin R. Garimella K. V. Maguire J. R. Hartl C. (2011). A framework for variation discovery and genotyping using next-generation DNA sequencing data. *Nat. Genet.* 43 491–498. 21478889 10.1038/ng.806 PMC3083463

[B20] Dewan R. Pemov A. Dutra A. S. Pak E. D. Edwards N. A. Ray-Chaudhury A. (2017). First insight into the somatic mutation burden of neurofibromatosis type 2-associated grade I and grade II meningiomas: a case report comprehensive genomic study of two cranial meningiomas with vastly different clinical presentation. *BMC Cancer* 17:127. 10.1186/s12885-017-3127-6 28193203 PMC5307647

[B21] Gautschi O. Milia J. Cabarrou B. Bluthgen M. V. Besse B. Smit E. F. (2015). Targeted Therapy for Patients with BRAF-Mutant Lung Cancer: results from the European EURAF Cohort. *J. Thorac. Oncol.* 10 1451–1457. 10.1097/jto.0000000000000625 26200454

[B22] Goodman A. M. Kato S. Bazhenova L. Patel S. P. Frampton G. M. Miller V. (2017). Tumor Mutational Burden as an Independent Predictor of Response to Immunotherapy in Diverse Cancers. *Mol. Cancer Ther.* 16 2598–2608. 10.1158/1535-7163.mct-17-0386 28835386 PMC5670009

[B23] Goutagny S. Yang H. W. Zucman-Rossi J. Chan J. Dreyfuss J. M. Park P. J. (2010). Genomic profiling reveals alternative genetic pathways of meningioma malignant progression dependent on the underlying NF2 status. *Clin. Cancer Res.* 16 4155–4164. 10.1158/1078-0432.ccr-10-0891 20682713

[B24] Gu M. J. Huang Q. C. Bao C. Z. Li Y. J. Li X. Q. Ye D. (2018). Attributable causes of colorectal cancer in China. *BMC Cancer* 18:38. 10.1186/s12885-017-3968-z 29304763 PMC5756355

[B25] Guinney J. Dienstmann R. Wang X. De Reynies A. Schlicker A. Soneson C. (2015). The consensus molecular subtypes of colorectal cancer. *Nat. Med.* 21 1350–1356. 26457759 10.1038/nm.3967 PMC4636487

[B26] Janku F. Connolly R. Lorusso P. De Jonge M. Vaishampayan U. Rodon J. (2015). Abstract C45: phase I study of WNT974, a first-in-class Porcupine inhibitor, in advanced solid tumors. *Mol. Cancer Ther.* 14 C45–C45.

[B27] Janku F. De Vos F. De Miguel M. Forde P. Ribas A. Nagasaka M. (2020). Abstract CT034: phase I study of WNT974 + spartalizumab in patients (pts) with advanced solid tumors. *Cancer Res.* 80 CT034–CT034.

[B28] Janssen A. Medema R. H. (2013). Genetic instability: tipping the balance. *Oncogene* 32 4459–4470. 10.1038/onc.2012.576 23246960

[B29] Jin Y. Bao H. Le X. Fan X. Tang M. Shi X. (2020). Distinct co-acquired alterations and genomic evolution during TKI treatment in non-small-cell lung cancer patients with or without acquired T790M mutation. *Oncogene* 39 1846–1859. 10.1038/s41388-019-1104-z 31754213

[B30] Jung Y. S. Park J. I. (2020). Wnt signaling in cancer: therapeutic targeting of Wnt signaling beyond beta-catenin and the destruction complex. *Exp. Mol. Med.* 52 183–191. 10.1038/s12276-020-0380-6 32037398 PMC7062731

[B31] Kinzler K. W. Vogelstein B. (1996). Lessons from hereditary colorectal cancer. *Cell* 87 159–170. 10.1016/s0092-8674(00)81333-1 8861899

[B32] Koboldt D. C. Zhang Q. Larson D. E. Shen D. Mclellan M. D. Lin L. (2012). VarScan 2: somatic mutation and copy number alteration discovery in cancer by exome sequencing. *Genome Res.* 22 568–576. 10.1101/gr.129684.111 22300766 PMC3290792

[B33] Koo B. K. Spit M. Jordens I. Low T. Y. Stange D. E. Van De Wetering M. (2012). Tumour suppressor RNF43 is a stem-cell E3 ligase that induces endocytosis of Wnt receptors. *Nature* 488 665–669. 10.1038/nature11308 22895187

[B34] Lee A. J. Endesfelder D. Rowan A. J. Walther A. Birkbak N. J. Futreal P. A. (2011). Chromosomal instability confers intrinsic multidrug resistance. *Cancer Res.* 71 1858–1870. 10.1158/0008-5472.can-10-3604 21363922 PMC3059493

[B35] Li H. Durbin R. (2009). Fast and accurate short read alignment with Burrows-Wheeler transform. *Bioinformatics* 25 1754–1760. 10.1093/bioinformatics/btp324 19451168 PMC2705234

[B36] Liu Y. Sethi N. S. Hinoue T. Schneider B. G. Cherniack A. D. Sanchez-Vega F. (2018). Comparative Molecular Analysis of Gastrointestinal Adenocarcinomas. *Cancer Cell* 33 721–735.e8. 29622466 10.1016/j.ccell.2018.03.010 PMC5966039

[B37] Loregger A. Grandl M. Mejias-Luque R. Allgauer M. Degenhart K. Haselmann V. (2015). The E3 ligase RNF43 inhibits Wnt signaling downstream of mutated beta-catenin by sequestering TCF4 to the nuclear membrane. *Sci. Signal.* 8:ra90. 10.1126/scisignal.aac6757 26350900

[B38] Maleki S. S. Rocken C. (2017). Chromosomal Instability in Gastric Cancer Biology. *Neoplasia* 19 412–420. 10.1016/j.neo.2017.02.012 28431273 PMC5397576

[B39] Malumbres M. (2011). Oncogene-induced mitotic stress: p53 and pRb get mad too. *Cancer Cell* 19 691–692. 10.1016/j.ccr.2011.05.023 21665141

[B40] Matano M. Date S. Shimokawa M. Takano A. Fujii M. Ohta Y. (2015). Modeling colorectal cancer using CRISPR-Cas9-mediated engineering of human intestinal organoids. *Nat. Med.* 21 256–262. 10.1038/nm.3802 25706875

[B41] Mermel C. H. Schumacher S. E. Hill B. Meyerson M. L. Beroukhim R. Getz G. (2011). GISTIC2.0 facilitates sensitive and confident localization of the targets of focal somatic copy-number alteration in human cancers. *Genome Biol.* 12:R41. 10.1186/gb-2011-12-4-r41 PMC3218867 21527027

[B42] Merten L. Agaimy A. Moskalev E. A. Giedl J. Kayser C. Geddert H. (2016). Inactivating Mutations of RB1 and TP53 Correlate With Sarcomatous Histomorphology and Metastasis/Recurrence in Gastrointestinal Stromal Tumors. *Am. J. Clin. Pathol.* 146 718–726. 10.1093/ajcp/aqw193 28028119

[B43] Mertz T. M. Baranovskiy A. G. Wang J. Tahirov T. H. Shcherbakova P. V. (2017). Nucleotide selectivity defect and mutator phenotype conferred by a colon cancer-associated DNA polymerase delta mutation in human cells. *Oncogene* 36 4427–4433. 10.1038/onc.2017.22 28368425 PMC5542868

[B44] Mirabelli C. K. Nusse R. Tuveson D. A. Williams B. O. (2019). Perspectives on the role of Wnt biology in cancer. *Sci. Signal.* 12:eaay4494. 10.1126/scisignal.aay4494 31289213

[B45] Mo S. Ma X. Li Y. Zhang L. Hou T. Han-Zhang H. (2020). Somatic POLE exonuclease domain mutations elicit enhanced intratumoral immune responses in stage II colorectal cancer. *J. Immunother. Cancer* 8:e000881. 10.1136/jitc-2020-000881 32859741 PMC7454238

[B46] Neumeyer V. Brutau-Abia A. Allgauer M. Pfarr N. Weichert W. Falkeis-Veits C. (2020). Loss of RNF43 Function Contributes to Gastric Carcinogenesis by Impairing DNA Damage Response. *Cell. Mol. Gastroenterol. Hepatol.* 11 1071–1094. 10.1016/j.jcmgh.2020.11.005 33188943 PMC7898035

[B47] Neumeyer V. Grandl M. Dietl A. Brutau-Abia A. Allgauer M. Kalali B. (2019). Loss of endogenous RNF43 function enhances proliferation and tumour growth of intestinal and gastric cells. *Carcinogenesis* 40 551–559. 10.1093/carcin/bgy152 30380024

[B48] Newman A. M. Bratman S. V. Stehr H. Lee L. J. Liu C. L. Diehn M. (2014). FACTERA: a practical method for the discovery of genomic rearrangements at breakpoint resolution. *Bioinformatics* 30 3390–3393. 10.1093/bioinformatics/btu549 25143292 PMC4296148

[B49] Olivier M. Eeles R. Hollstein M. Khan M. A. Harris C. C. Hainaut P. (2002). The IARC TP53 database: new online mutation analysis and recommendations to users. *Hum. Mutat.* 19 607–614. 10.1002/humu.10081 12007217

[B50] Pages F. Berger A. Camus M. Sanchez-Cabo F. Costes A. Molidor R. (2005). Effector memory T cells, early metastasis, and survival in colorectal cancer. *N. Engl. J. Med.* 353 2654–2666. 10.1056/nejmoa051424 16371631

[B51] Pages F. Galon J. Fridman W. H. (2008). The essential role of the in situ immune reaction in human colorectal cancer. *J. Leukoc Biol.* 84 981–987. 10.1189/jlb.1107773 18559950

[B52] Pikor L. Thu K. Vucic E. Lam W. (2013). The detection and implication of genome instability in cancer. *Cancer Metastasis Rev.* 32 341–352. 10.1007/s10555-013-9429-5 23633034 PMC3843371

[B53] Pujade-Lauraine E. Ledermann J. A. Selle F. Gebski V. Penson R. T. Oza A. M. (2017). Olaparib tablets as maintenance therapy in patients with platinum-sensitive, relapsed ovarian cancer and a BRCA1/2 mutation (SOLO2/ENGOT-Ov21): a double-blind, randomised, placebo-controlled, phase 3 trial. *Lancet Oncol.* 18 1274–1284. 28754483 10.1016/S1470-2045(17)30469-2

[B54] Rayner E. Van Gool I. C. Palles C. Kearsey S. E. Bosse T. Tomlinson I. (2016). A panoply of errors: polymerase proofreading domain mutations in cancer. *Nat. Rev. Cancer* 16 71–81. 10.1038/nrc.2015.12 26822575

[B55] Robert C. Karaszewska B. Schachter J. Rutkowski P. Mackiewicz A. Stroiakovski D. (2015). Improved overall survival in melanoma with combined dabrafenib and trametinib. *N. Engl. J. Med.* 372 30–39. 25399551 10.1056/NEJMoa1412690

[B56] Roylance R. Endesfelder D. Gorman P. Burrell R. A. Sander J. Tomlinson I. (2011). Relationship of extreme chromosomal instability with long-term survival in a retrospective analysis of primary breast cancer. *Cancer Epidemiol. Biomarkers Prev.* 20 2183–2194. 10.1158/1055-9965.epi-11-0343 21784954 PMC3199437

[B57] Ryu M.-H. Lee H. Kim T. W. Chang H.-M. Kim J.-S. Kim W.-K. (2004). p53 mutations in gastrointestinal stromal tumor: pattern and prognostic significance. *J. Clin. Oncol.* 22 9026–9026. 10.1200/jco.2004.22.14_suppl.9026

[B58] Sanchez-Vega F. Mina M. Armenia J. Chatila W. K. Luna A. La K. C. (2018). Oncogenic Signaling Pathways in the Cancer Genome Atlas. *Cell* 173 321–337.e10. 29625050 10.1016/j.cell.2018.03.035 PMC6070353

[B59] Shu Y. Wu X. Tong X. Wang X. Chang Z. Mao Y. (2017). Circulating Tumor DNA Mutation Profiling by Targeted Next Generation Sequencing Provides Guidance for Personalized Treatments in Multiple Cancer Types. *Sci. Rep.* 7:583. 10.1038/s41598-017-00520-1 PMC5428730 28373672

[B60] Snyder A. Makarov V. Merghoub T. Yuan J. Zaretsky J. M. Desrichard A. (2014). Genetic basis for clinical response to CTLA-4 blockade in melanoma. *N. Engl. J. Med.* 371 2189–2199. 10.1056/nejmoa1406498 25409260 PMC4315319

[B61] Sun S. Q. Mashl R. J. Sengupta S. Scott A. D. Wang W. Batra P. (2018). Database of evidence for precision oncology portal. *Bioinformatics* 34 4315–4317. 10.1093/bioinformatics/bty531 30535306 PMC6289129

[B62] Thompson S. Bakhoum S. Compton D. Thompson S. Bakhoum S. Compton D. (2010). Mechanisms of chromosomal instability. *Curr. Biol.* 20 R285–R295. 20334839 10.1016/j.cub.2010.01.034 PMC3781365

[B63] Thompson S. L. Compton D. A. (2010). Proliferation of aneuploid human cells is limited by a p53-dependent mechanism. *J. Cell Biol.* 188 369–381. 10.1083/jcb.200905057 20123995 PMC2819684

[B64] Turajlic S. Xu H. Litchfield K. Rowan A. Horswell S. Chambers T. (2018). Deterministic Evolutionary Trajectories Influence Primary Tumor Growth: TRACERx Renal. *Cell* 173 595–610.e11. 29656894 10.1016/j.cell.2018.03.043 PMC5938372

[B65] Vogelstein B. Fearon E. R. Hamilton S. R. Kern S. E. Preisinger A. C. Leppert M. (1988). Genetic alterations during colorectal-tumor development. *N. Engl. J. Med.* 319 525–532. 10.1056/nejm198809013190901 2841597

[B66] Wang K. Li M. Hakonarson H. (2010). ANNOVAR: functional annotation of genetic variants from high-throughput sequencing data. *Nucleic Acids Res.* 38:e164. 10.1093/nar/gkq603 20601685 PMC2938201

[B67] Wardell C. P. Fujita M. Yamada T. Simbolo M. Fassan M. Karlic R. (2018). Genomic characterization of biliary tract cancers identifies driver genes and predisposing mutations. *J. Hepatol.* 68 959–969. 10.1016/j.jhep.2018.01.009 29360550

[B68] Wozniak A. Rutkowski P. Piskorz A. Ciwoniuk M. Osuch C. Bylina E. (2012). Prognostic value of KIT/PDGFRA mutations in gastrointestinal stromal tumours (GIST): polish Clinical GIST Registry experience. *Ann. Oncol.* 23 353–360. 10.1093/annonc/mdr127 21527588

[B69] Yaeger R. Chatila W. K. Lipsyc M. D. Hechtman J. F. Cercek A. Sanchez-Vega F. (2018). Clinical Sequencing Defines the Genomic Landscape of Metastatic Colorectal Cancer. *Cancer Cell* 33 125–136.e3. 29316426 10.1016/j.ccell.2017.12.004 PMC5765991

[B70] Yang Z. Yang N. Ou Q. Xiang Y. Jiang T. Wu X. (2018). Investigating Novel Resistance Mechanisms to Third-Generation EGFR Tyrosine Kinase Inhibitor Osimertinib in Non-Small Cell Lung Cancer Patients. *Clin. Cancer Res.* 24 3097–3107. 10.1158/1078-0432.ccr-17-2310 29506987

[B71] Yu J. Yusoff P. A. M. Woutersen D. T. J. Goh P. Harmston N. Smits R. (2020). The Functional Landscape of Patient-Derived RNF43 Mutations Predicts Sensitivity to Wnt Inhibition. *Cancer Res.* 80 5619–5632. 10.1158/0008-5472.can-20-0957 33067269

[B72] Zack T. I. Schumacher S. E. Carter S. L. Cherniack A. D. Saksena G. Tabak B. (2013). Pan-cancer patterns of somatic copy number alteration. *Nat. Genet.* 45 1134–1140. 24071852 10.1038/ng.2760 PMC3966983

