# Supplementary material for: Genomic Profiling Reveals the Molecular Landscape of Gastrointestinal Tract Cancers in Chinese Patients
Source: Front Genet. 2021 Sep 14;12:608742. doi: 10.3389/fgene.2021.608742 (PMC8478156; doi:10.3389/fgene.2021.608742)
Supplement: Supplementary file 1 [file Data_Sheet_1.DOCX]

Supplementary Material

**Supplementary Table 1.** Comparison of clinical characteristics between our colorectal cancer cohort and the MSKCC colorectal cancer cohort

|  | **Cohort in this study** | | **MSKCC** | |
| --- | --- | --- | --- | --- |
| **Characteristics** | **Patient No. = 207** | **%** | **Patient No. = 985** | **%** |
| **Age (years)** |  |  |  |  |
| Median | 59 | - | 54 | - |
| Range | 26-87 | - | 13-93 | - |
| **Gender** |  |  |  |  |
| Female | 86 | 41.5 | 441 | 44.8 |
| Male | 121 | 58.5 | 515 | 52.3 |
| **Clinical stage** |  |  |  |  |
| I-II | 13 | 4.9 | 150 | 15.2 |
| III | 20 | 9.5 | 227 | 23.0 |
| IV | 161 | 80.3 | 608 | 61.7 |
| Unknown | 13 | 5.3 | -- | -- |
| **Primary tumor location** |  |  |  |  |
| Right-sided | 48 | 22.3 | 298 | 30.2 |
| Left-sided | 149 | 72.0 | 678 | 68.5 |
| Unknown | 10 | 4.7 | 12 | 1.3 |

**Supplementary Table 2.** Clinical characteristics of the gastric cancer cohort

| **Characteristics** | **Patient No. = 144** | **%** |
| --- | --- | --- |
| **Age (years)** |  |  |
| Median | 60 | - |
| Range | 32-81 | - |
| **Gender** |  |  |
| Female | 38 | 26.4 |
| Male | 106 | 73.6 |
| **Histological classification** |  |  |
| Adenocarcinoma | 73 | 50.7 |
| Squamous carcinoma | 1 | 0.7 |
| NET | 4 | 2.8 |
| Unknown | 66 | 45.8 |
| **Clinical stage** |  |  |
| I-II | 7 | 4.9 |
| III | 25 | 17.4 |
| IV | 99 | 68.8 |
| Unknown | 13 | 9.0 |
| **Primary tumor location** |  |  |
| Cardia | 27 | 17.4 |
| Fundus | 8 | 5.6 |
| Body | 58 | 40.3 |
| Pylorus | 27 | 18.8 |
| Duodenum | 3 | 2.1 |
| Unknown | 23 | 16.0 |

**Supplementary Table 3.** Clinical characteristics of the gallbladder and bile duct cancer cohorts

| **Characteristics** | **Patient No. = 14** | **%** |
| --- | --- | --- |
| **Age (years)** |  |  |
| Median | 53 | - |
| Range | 43-78 | - |
| **Gender** |  |  |
| Female | 8 | 57.1 |
| Male | 6 | 42.9 |
| **Clinical stage** |  |  |
| II-III | 2 | 14.3 |
| IV | 8 | 57.1 |
| Unknown | 4 | 28.6 |
| **Location** |  |  |
| Gallbladder | 5 | 35.7 |
| Biliary | 9 | 64.3 |

**Supplementary Table 4.** Clinical characteristics of the pancreatic cancer cohort

| **Characteristics** | **Patient No. = 27** | **Percentage (%)** |
| --- | --- | --- |
| **Age (years)** |  |  |
| Median | 60 | - |
| Range | 38-78 | - |
| **Gender** |  |  |
| Female | 6 | 31.1 |
| Male | 21 | 68.9 |
| **Clinical stage** |  |  |
| II-III | 9 | 33.3 |
| IV | 12 | 44.4 |
| Unknown | 6 | 22.2 |
| **Location** |  |  |
| Head | 2 | 7.4 |
| Body | 7 | 25.9 |
| Tail | 2 | 7.4 |
| Unknown | 16 | 59.3 |

**Supplementary Table 5.** Clinical characteristics of the GIST cancer cohort

| **Characteristics** | **Patient No. = 22** | **Percentage (%)** |
| --- | --- | --- |
| **Age (years)** |  |  |
| Median | 64 | - |
| Range | 24-83 | - |
| **Gender** |  |  |
| Female | 10 | 45.5 |
| Male | 12 | 54.5 |
| **Location** |  |  |
| Duodenum | 3 | 13.6 |
| Ileocecal | 1 | 4.5 |
| Small intestine | 2 | 9.1 |
| Stomach | 4 | 18.2 |
| Unknown | 12 | 54.5 |


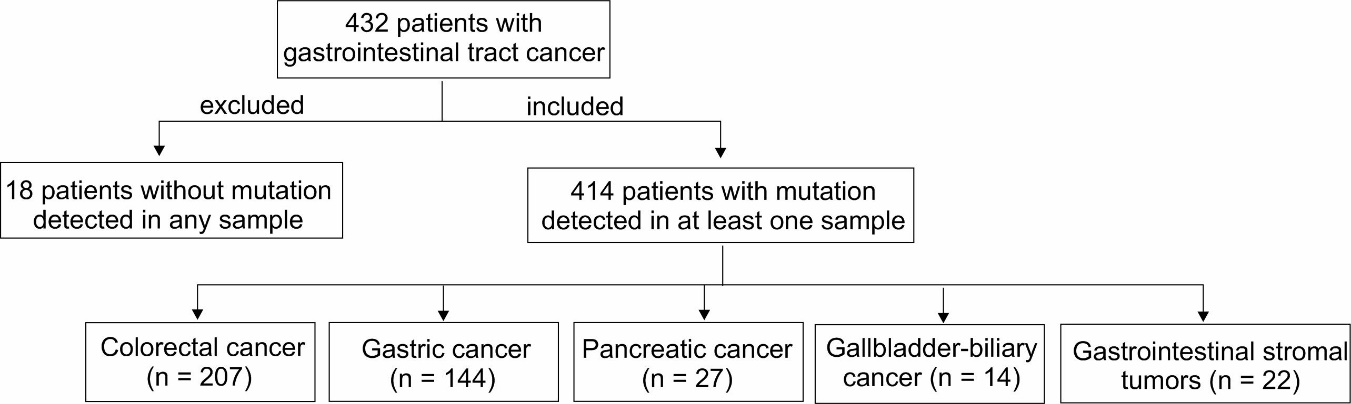


**Suppl. Fig. 1** Patient cohorts in different cancers


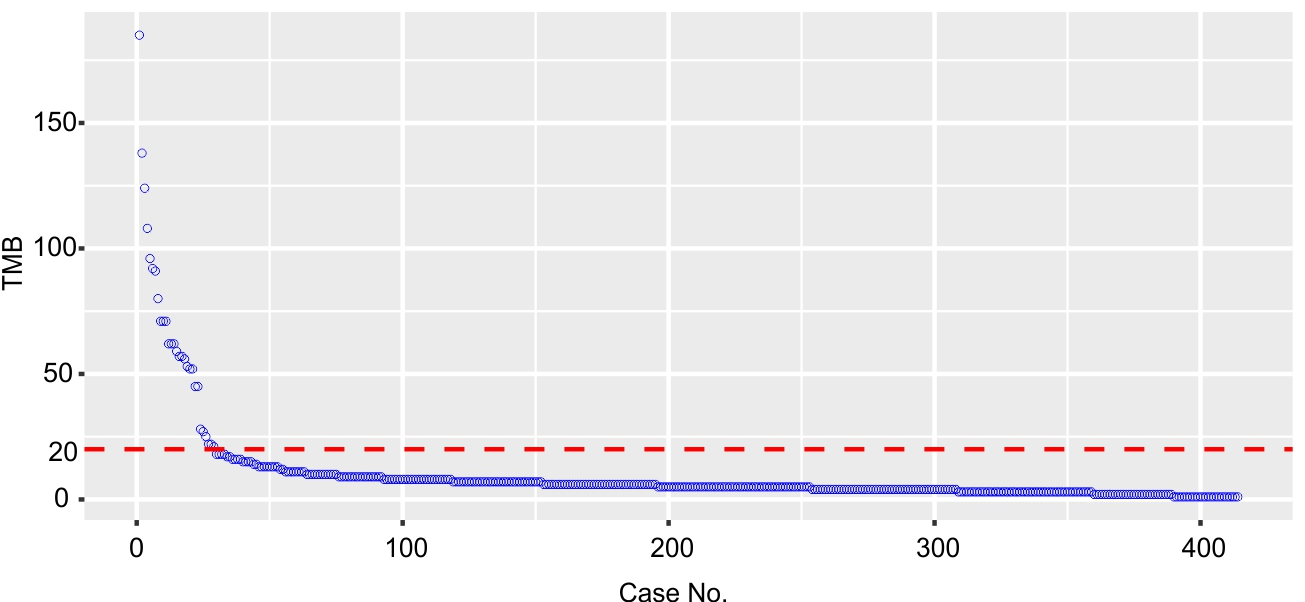


**Suppl. Fig. 2** TMB in all patients. Red dotted line indicates the cut-off for hyper-mutated tumors (TMB > 20).


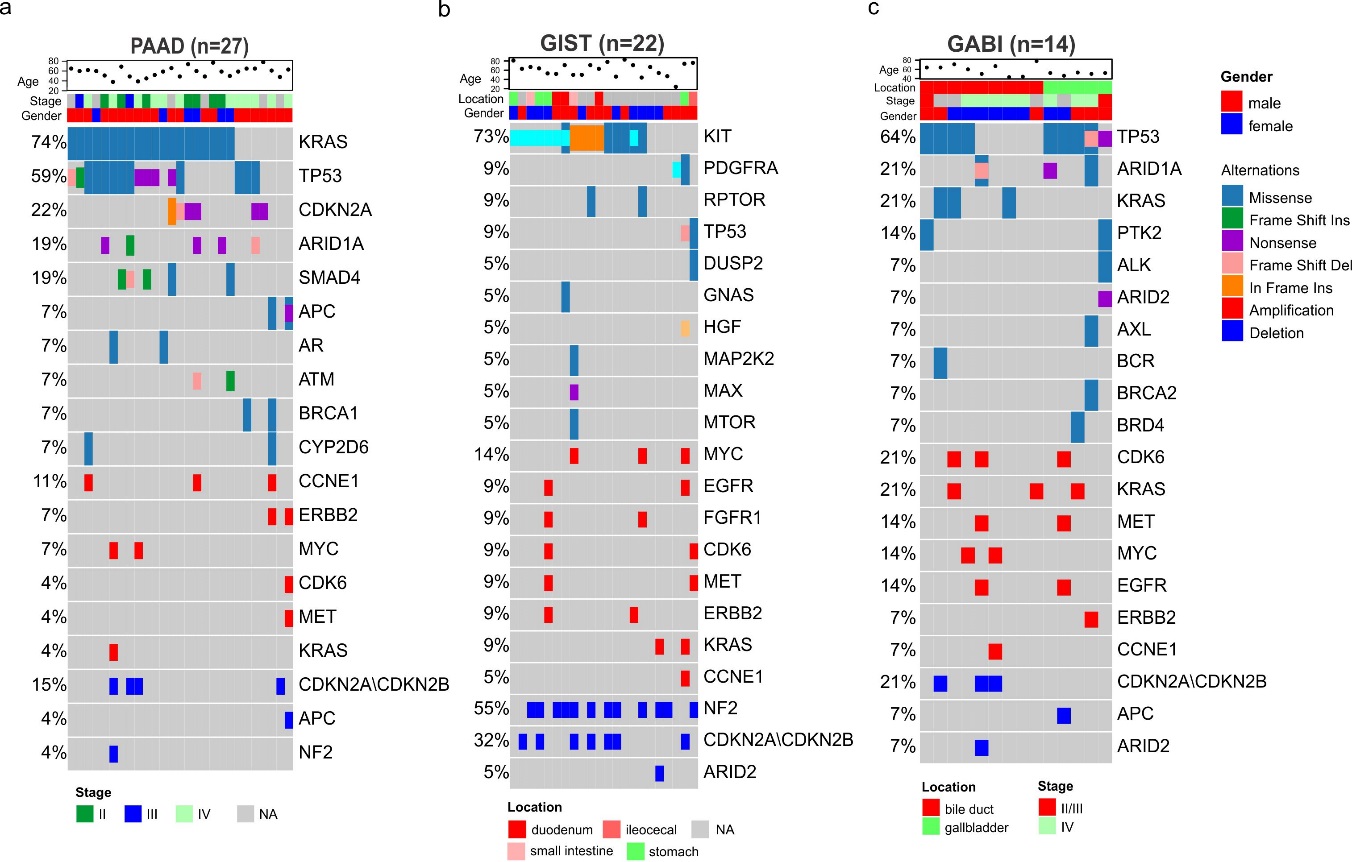


**Suppl. Fig. 3** Co-mutation plot for PAAD (a), GIST (b), and GABI (c) cancers.


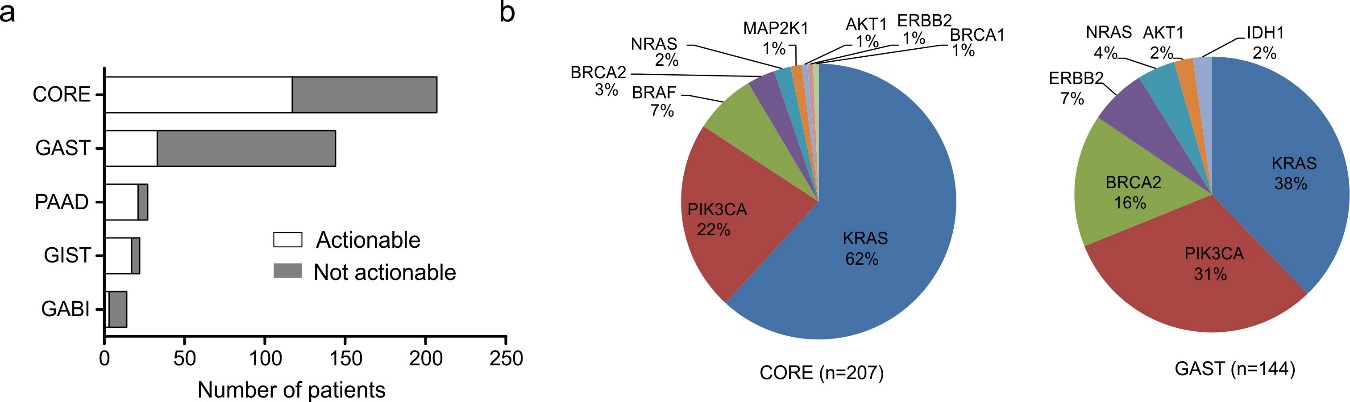


**Suppl. Fig. 4** Actionable mutations in different GI cancers. a) The proportion of patients with actionable mutations in each cancer. b) The specific genes that are targetable in CORE and GAST.
